# Stacked Polarizing Elements for Controlling Parameters of Surface Relief Gratings Written in Photosensitive Materials

**DOI:** 10.3390/s24041166

**Published:** 2024-02-10

**Authors:** Alexey P. Porfirev, Svetlana N. Khonina, Nikolay A. Ivliev, Denis P. Porfirev, Nikolay L. Kazanskiy

**Affiliations:** 1 Image Processing Systems Institute of RAS—Branch of the FSRC “Crystallography and Photonics” RAS, Molodogvardeyskaya Str. 151, 443001 Samara, Russia; porfirev.alexey@ipsiras.ru (A.P.P.); khonina@ipsiras.ru (S.N.K.); ivlievn@gmail.com (N.A.I.); porfirev.dp@ssau.ru (D.P.P.); 2 Image Processing Systems Institute, National Research Centre “Kurchatov Institute”, Molodogvardeyskaya Str. 151, 443001 Samara, Russia; 3 Scientific Research Laboratory of Automated Systems of Scientific Research (SRL-35), Samara National Research University, Moskovskoye Shosse 34, 443086 Samara, Russia; 4 The Center for Laboratory Astrophysics, Samara Branch of P.N. Lebedev Physical Institute of the Russian Academy of Sciences, 221 Novo-Sadovaya Str., 443011 Samara, Russia

**Keywords:** surface relief gratings, depolarizer, *q*-plate, polarization gratings, chalcogenide glasses

## Abstract

Photosensitive materials are widely used for the direct fabrication of surface relief gratings (SRGs) without the selective etching of the material. It is known that the interferometric approach makes it possible to fabricate SRGs with submicron and even subwavelength periods. However, to change the period of the written SRGs, it is necessary to change the convergence angle, shift a sample, and readjust the interferometric setup. Recently, it was shown that structured laser beams with predetermined, periodically modulated polarization distributions can also be used to fabricate SRGs. A structured laser beam with the desired polarization distribution can be formed with just one polarizing optical element—for example, the so-called depolarizer, a patterned micro-retarder array. The use of such stacked elements makes it possible to directly control the modulation period of the polarization of the generated laser beam. We show that this approach allows one to fabricate SRGs with submicron periods. Moreover, the addition of *q*-plates, elements effectively used to generate cylindrical vector beams with polarization singularities, allows the efficient formation of fork polarization gratings (FPGs) and the fabrication of higher-order fork-shaped SRGs. Full control of the parameters of the generated FPGs is possible. We demonstrate the formation of FPGs of higher orders (up to 12) by only adding first- and second-order *q*-plates and half-wave plates to the depolarizers. In this work, we numerically and experimentally study the parameters of various types of SRGs formed using these stacked polarizing elements and show the significant potential of this method for the laser processing of photosensitive materials, which often also serve as polarization sensors.

## 1. Introduction

Surface relief gratings (SRGs) are widely used in spectroscopy [1,2,3,4], laser beam splitting [5,6], laser material processing [7], and solar cells [8,9]. Subwavelength versions of SRGs are used for polarization transformation and the generation of vector optical vortex beams [10,11,12,13,14]. The most common way to manufacture SRGs is the interferometric approach. The interference of two laser beams generates a periodically modulated intensity distribution for the processing of materials [15,16,17]. Laser-induced periodic surface structures (LIPSSs) formed using pulsed laser radiation [18,19] are used for the fabrication of subwavelength SRGs. To change the period of the formed periodic relief in these cases, it is necessary to change the wavelength of the illuminating laser radiation or change the parameters of the elements in the optical laser-writing setup. This is possible by changing the convergence angle of the superimposed laser beams in the interferometric setup or by using micro-objectives with different magnifications and numerical apertures. In our recent work, we proposed the use of the so-called depolarizer for the fabrication of SRGs in thin films of photosensitive chalcogenide glasses (CGs) [20]. Depolarizers are patterned micro-retarder arrays capable of converting the initial linear polarization of a laser beam into a periodically modulated polarization distribution [21]. It is well known that photosensitive materials can change their structures under the action of laser radiation, depending not only on the amplitude but also on the polarization distribution of the radiation [22]. Because of this, we have shown the possibility of controlling the relief height, the period of the formed SRGs, and the orientation of the grating grooves using a single depolarizer. However, the proposed approach had a certain limitation regarding the period of fabricated SRGs—the minimum period of the fabricated structures was only 4 µm. To overcome this limitation, we propose the use of a combination of two depolarizers. Each of these depolarizers performs the same polarization transformation; however, the rotation of one of them relative to the other leads to a change in the polarization modulation period in the transformed laser beam. Note, the generated intensity distribution remains the same. The period of these changes can either increase or decrease depending on the angle between the axes of the depolarizers. In the experimental testing, we applied thin films of nano-multilayer structures (NMLSs) based on the CGs’ As_2_S_3_ and a-Se [23,24]. For the laser writing of the SRGs, we used a laser radiation with a wavelength of 532 nm, which is in a region of a significant decrease in the transparency of the film (450–650 nm) because of an increase in the absorption.

We have also explored the possibility of using other polarizing elements in combination with depolarizers. Combinations of a depolarizer and *q*-plates of different orders have been proposed to form fork-shaped polarization gratings (FPGs) [25]. Among the useful applications of FPGs are laser beam generation with the orbital angular momentum (OAM) and mode transformation [26,27]. It is well known that *q*-plates allows one to generate the so-called cylindrical vector beams (CVBs), beams with polarization singularities [28]. Our results show that the use of depolarizers combined with *q*-plates of the *q* = 1/2 and *q* = 1 orders leads to the formation of second- and fourth-order FPGs. These FPGs were effectively used for the laser processing of NMLSs based on CGs. In addition, the possibility of forming FPGs of the sixth, eighth, tenth, and twelfth orders using a combination of a depolarizer, *q*-plates with the orders *q* = 1/2 and 1, and half-wave plates was demonstrated. Thus, the combinations of low-order *q*-plates, depolarizers, and half-wave plates can be used for the effective generation of higher-order FPGs, which can be used for the laser patterning of thin films of photosensitive materials and fabrication fork-shaped SRGs [29]. Fork-shaped SRGs are well-studied elements for the generation of optical vortex (OV) beams with OAM [14,30,31,32,33]. Such elements are widely used in modern applications [34] and make it possible to generate OVs in the extreme ultraviolet region [35], as well as realize a white-light vortex coronagraph with sub-resolution detection [36].

## 2. Methods

### 2.1. Two Stacked Depolarizers

**Figure 1:**
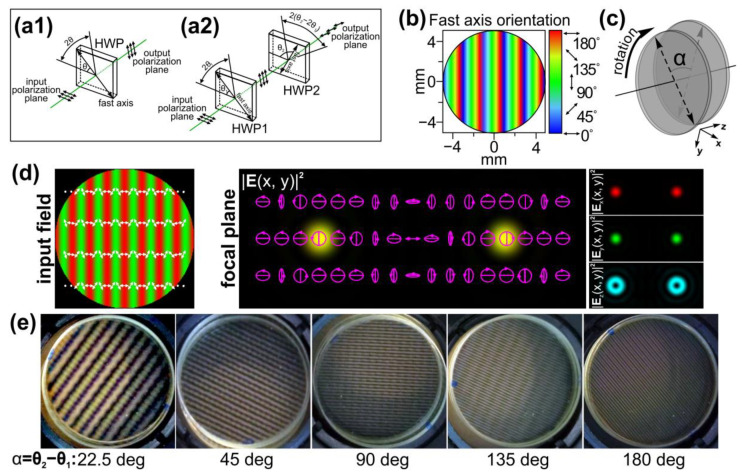
Polarization transformations carried out by (**a1**) a single half-wave plate and (**a2**) two stacked half-wave plates. (**b**) Fast-axis orientation of the depolarizer used in the study. (**c**) Schematic of the proposed principle for controlling the polarization modulation period using a double depolarizer–rotation of one of the used depolarizers relative to the other. (**d**) Focusing of a light field created by a linear polarization grating formed by a depolarizer. The three components of the electric field (red—*x*-component; green—*y*-component; and blue—*z*-component) of the input field and the total intensity distribution formed in the focal plane are shown. The arrows indicate local polarization directions. The ellipses with arrows are polarization ellipses. The size of the input field distributions is 200 × 200 μm. (**e**) Crossed-polarizer images of two stacked depolarizers obtained with various angle differences (α) between their axes.

The depolarizer used in our study is an array of wave plates with varying retardation and fast-axis orientation imprinted in the liquid crystal polymer (LCP) material [37]. It is well known that a wave plate can be used as a continuously adjustable polarization rotator; the polarization direction of the transmitted linearly polarized light is rotated by a double angle between the axis of the plate and the polarization direction of the incident light (see Figure 1(a1)). In terms of the Jones calculus, such a transformation of *x*-polarized light can be defined as follows [38]:

(1) $$J_{1}=\left[\begin{array}{cc} \cos2\theta_{1} & \sin2\theta_{1}\\ \sin2\theta_{1} & -\cos2\theta_{1} \end{array}\right]\left[\begin{array}{c} 1\\ 0 \end{array}\right]=\left[\begin{array}{c} \cos2\theta_{1}\\ \sin2\theta_{1} \end{array}\right],$$

where θ_1_ is the angle between the fast axis of the plate and the horizontal.

If a second half-wave plate is used, then the Jones vector of the laser beam at the output of the second plate with the axis rotated by θ_2_ relative to the horizontal has the following form:

(2) $$J_{2}=\left[\begin{array}{cc} \cos2\theta_{2} & \sin2\theta_{2}\\ \sin2\theta_{2} & -\cos2\theta_{2} \end{array}\right]\left[\begin{array}{c} \cos2\theta_{1}\\ \sin2\theta_{1} \end{array}\right]=\left[\begin{array}{c} \cos\left(2\theta_{2}-2\theta_{1}\right)\\ \sin\left(2\theta_{2}-2\theta_{1}\right) \end{array}\right].$$

Thus, the polarization plane of the linearly polarized light passing through two stacked half-wave plates is rotated by the angle 2θ_2_ − 2θ_1_. Compared to the case of using one half-wave plate, the second plate additionally rotates the polarization plane of the light transmitted through the first plate by an angle of 2(θ_2_ − 2θ_1_) (see Figure 1(a2)).

The patterns of varying the retardation and fast-axis orientation imprinted in depolarizers make it possible to realize different transformations of the initial uniform linear polarization of the incident radiation. Because of this, it is possible to generate various patterns of the polarization distribution. For widely used commercially available depolarizers [21], the angle of the fast axis increases by a constant value across each consecutive strip section in the element (see Figure 1b). Let us assume that the angle of the axis increases along the *x*-axis. Then, the Jones matrix of such an element is defined as follows:


(3) $$J_{dpe}=\left[\begin{array}{cc} \cos2\theta (x) & \sin2\theta (x)\\ \sin2\theta (x) & -\cos2\theta (x) \end{array}\right].$$

A linear polarization grating formed from an initial linearly polarized Gaussian beam using this element acts like a conventional linear diffraction grating. Focusing of the generated light-field results in the generation of two light spots in the ±1 diffraction orders. However, these light spots have orthogonal circular polarizations (see Figure 1d). Therefore, in the regions before and after the focal plane, the interference of two Gaussian beams with right and left circular polarization occurs. As mentioned in [23], the interference of two such circularly polarized laser beams is widely used for the fabrication of linear SRGs, including subwavelength SRGs.

The Jones matrix of two stacked depolarizers with the distributions of the angles of the fast axis (θ_1_(*x*) and θ_2_(*x*) for the first and second element, respectively) has the following form:

(4) $$J_{dd}=\left[\begin{array}{cc} \cos2\left[\theta_{2}\left(x\right)-\theta_{1}\left(x\right)\right] & \sin2\left[\theta_{1}\left(x\right)-\theta_{2}\left(x\right)\right]\\ \sin2\left[\theta_{2}\left(x\right)-\theta_{1}\left(x\right)\right] & \cos2\left[\theta_{1}\left(x\right)-\theta_{2}\left(x\right)\right] \end{array}\right].$$

With a single depolarizer, the polarization transformation from a linear polarization into a spatially variant polarization occurs regardless of the element’s rotation angle. However, with two stacked depolarizers, if the orientations of the elements coincide, then, as follows from Equation (4), the Jones matrix is transformed into the following:

(5) $$J_{dd}=\left[\begin{array}{cc} 1 & 0\\ 0 & 1 \end{array}\right]$$

and there is no polarization modulation of the incident *x*-linearly polarized light. The rotation of the second depolarizer relative to the first one leads to the transformation of the total Jones matrix and results in a change in the period of the polarization modulation of the transmitted light (see Figure 1c,d).

Let us assume that θ(*x*) has a linear dependence of the angle on the coordinate:

(6) $$\theta \left(x\right)=\theta_{0}\frac{x}{T},$$

where *T* is a normalization constant and θ_0_ is the parameter corresponding to the rate of change of the angle. In the case of identical depolarizers rotated by an angle α = θ_2_ − θ_1_:

(7) $$\theta_{1}\left(x\right)=\theta_{0}\frac{x}{T},$$

(8) $$\theta_{2}\left(x\right)=\theta_{0}\frac{x\cos\alpha }{T}.$$

Taking into account that the local angle of rotation of the initial linearly polarized field by two stacked depolarizers is determined as 2(θ_2_ − 2θ_1_) + 2θ_1_ = 2(θ_2_ − θ_1_), then the period of the shaped polarization modulation is defined as follows:

(9) $$d_{dd}=\frac{\pi }{\theta_{0}}T\left[\frac{1}{1-\cos\alpha }\right]=\frac{d_{dpe}}{\eta },$$

where *d_dpe_* is the period of the polarization modulation shaped with a single depolarizer and η is the ratio of the modulation periods formed by two stacked depolarizers and one depolarizer. Thus, the period for two stacked elements decreases by a factor of η = 1 − cosα. The maximum decrease, η = 2, is in the case when α = π.

This feature allows one to dynamically control the period of SRGs written in photosensitive materials. An additional advantage of using stacked depolarizers is that the minimum achieved period can be significantly reduced. It should be noted that the polarization distributions generated in both cases, both when using a single depolarizer and when using stacked depolarizers, have profiles that coincide with the case of the interference of two circularly polarized laser beams. Such light fields are widely used for the fabrication of SRGs in CGs and azopolymers [20]. It is very convenient to use this approach for controlling the precision laser processing of such materials.

### 2.2. Combinations of a Depolarizer and Higher-Order q-Plates

**Figure 2:**
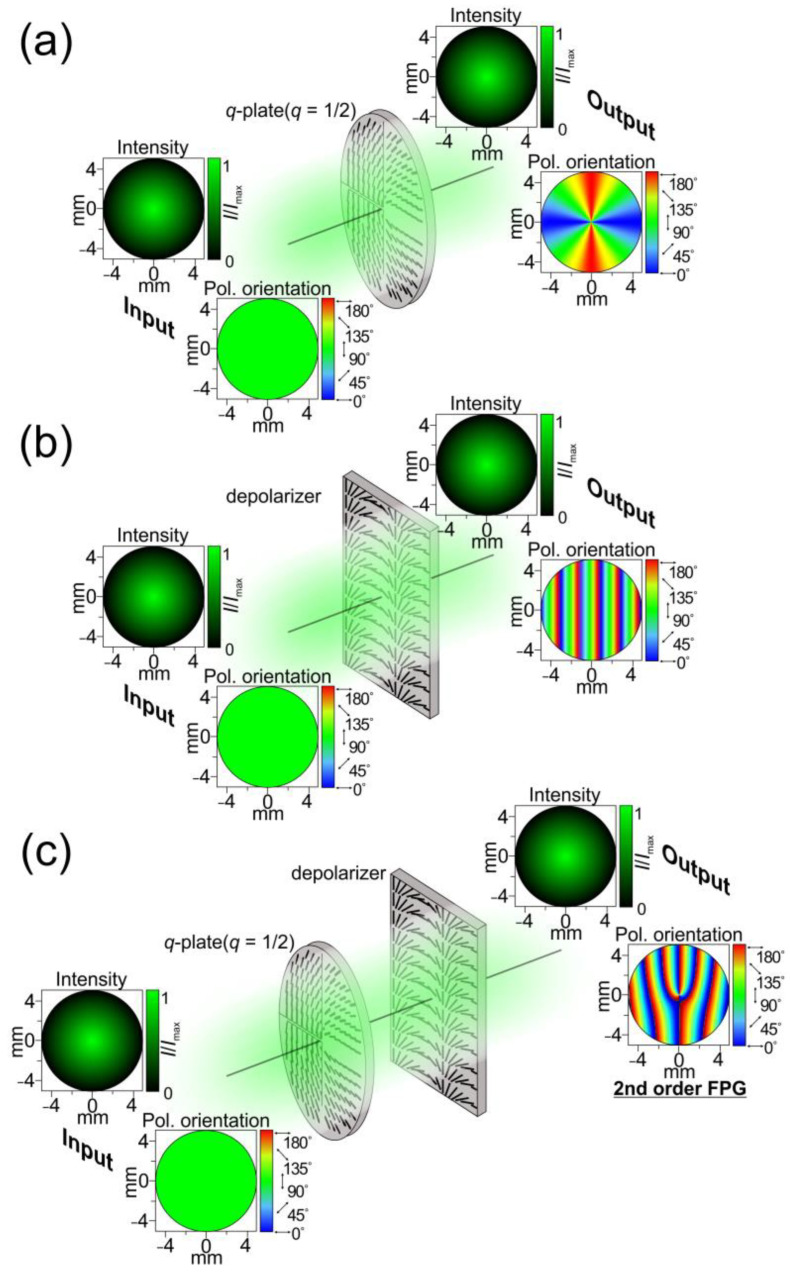
Schematic representation of the light-field transformations carried out by a single *q*-plate (*q* = ½) (**a**), a single depolarizer (**b**), and their combination (**c**).

Let us study combinations of a depolarizer with other polarizing elements to generate a structured polarized light field. Figure 2 shows an example of using the combination of a depolarizer and a *q*-plate with an order of *q*. The Jones matrix of a *q*-plate in the polar coordinates (*r*, ϕ) is defined as follows [39]:

(10) $$J_{q}=\left[\begin{array}{cc} \cos\left(2q\varphi \right) & \sin\left(2q\varphi \right)\\ \sin\left(2q\varphi \right) & -\cos\left(2q\varphi \right) \end{array}\right].$$

A *q*-plate of the order *q* allows one to form a CVB of the order 2*q*. The combination of such an element and a depolarizer with a linear dependence of the angle of the axis along the *x* axis in the form α*x* has the following Jones matrix:

(11) $$\begin{array}{l} J_{q\_fork}=\left[\begin{array}{cc} \cos\left(\alpha x\right) & \sin\left(\alpha x\right)\\ \sin\left(\alpha x\right) & -\cos\left(\alpha x\right) \end{array}\right]\left[\begin{array}{cc} \cos\left(2q\varphi \right) & \sin\left(2q\varphi \right)\\ \sin\left(2q\varphi \right) & -\cos\left(2q\varphi \right) \end{array}\right]=\\ =\left[\begin{array}{cc} \cos\left(\alpha x\right)\cos\left(2q\varphi \right)+\sin\left(\alpha x\right)\sin\left(2q\varphi \right) & \cos\left(\alpha x\right)\sin\left(2q\varphi \right)-\sin\left(\alpha x\right)\cos\left(2q\varphi \right)\\ \sin\left(\alpha x\right)\cos\left(2q\varphi \right)-\cos\left(\alpha x\right)\sin\left(2q\varphi \right) & \sin\left(\alpha x\right)\sin\left(2q\varphi \right)+\cos\left(\alpha x\right)\cos\left(2q\varphi \right) \end{array}\right] \end{array}$$

or uses the well-known trigonometric relations:

(12) $$J_{q\_fork}=\left[\begin{array}{cc} \cos\left(\alpha x-2q\varphi \right) & -\sin\left(\alpha x-2q\varphi \right)\\ \sin\left(\alpha x-2q\varphi \right) & \cos\left(\alpha x-2q\varphi \right) \end{array}\right].$$

In the case of an incident *x*-linearly polarized light field, this corresponds to the following Jones vector of the transformed output light field:

(13) $$J_{q\_fork}^{X-lin}=\left[\begin{array}{cc} \cos\left(\alpha x-2q\varphi \right) & -\sin\left(\alpha x-2q\varphi \right)\\ \sin\left(\alpha x-2q\varphi \right) & \cos\left(\alpha x-2q\varphi \right) \end{array}\right]\left(\begin{array}{c} 1\\ 0 \end{array}\right)=\left(\begin{array}{c} \cos\left(\alpha x-2q\varphi \right)\\ \sin\left(\alpha x-2q\varphi \right) \end{array}\right).$$

Equation (13) can be represented as follows:

(14) $$\begin{array}{l} J_{q\_fork}^{X-lin}=\left(\begin{array}{c} \cos\left(\alpha x-2q\varphi \right)\\ \sin\left(\alpha x-2q\varphi \right) \end{array}\right)=\frac{1}{2}\left(\begin{array}{c} \exp\left[i\left(\alpha x-2q\varphi \right)\right]+\exp\left[-i\left(\alpha x-2q\varphi \right)\right]\\ -i\exp\left[i\left(\alpha x-2q\varphi \right)\right]+i\exp\left[-i\left(\alpha x-2q\varphi \right)\right] \end{array}\right)=\\ =\frac{1}{2}\exp\left[i\left(\alpha x-2q\varphi \right)\right]\left(\begin{array}{c} 1\\ -i \end{array}\right)+\frac{1}{2}\exp\left[-i\left(\alpha x-2q\varphi \right)\right]\left(\begin{array}{c} 1\\ i \end{array}\right)=\\ =\frac{1}{2}\exp\left(i\alpha x\right)\exp\left(-i2q\varphi \right)\left(\begin{array}{c} 1\\ -i \end{array}\right)+\frac{1}{2}\exp\left(-i\alpha x\right)\exp\left(i2q\varphi \right)\left(\begin{array}{c} 1\\ i \end{array}\right). \end{array}$$

The field defined by Equation (14) allows one to form two OV beams of the orders ±2*q* with orthogonal circular polarizations in ±1 diffraction orders in the focal plane.

**Figure 3:**
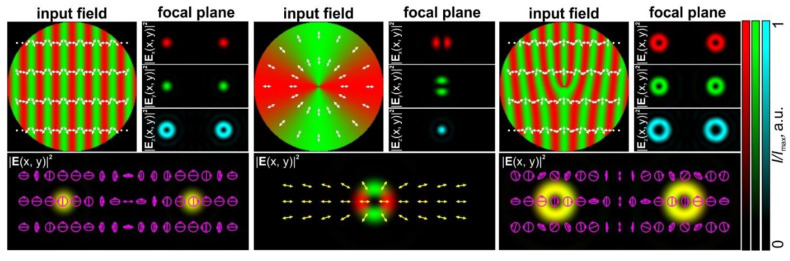
Focusing of light fields generated by a linear polarization grating formed by a depolarizer (**left**), a first-order CVB formed by a *q*-plate (*q* = 1/2), and an FPG formed by a combination of the *q*-plate and depolarizer. Three components of the electric field (red—*x*-component; green—*y*-component; and blue—*z*-component) and the total intensity distribution formed in the Fourier plane are shown. The arrows indicate local polarization directions. The ellipses with arrows are polarization ellipses. The size of the input field distributions is 200 × 200 μm.

Figure 3 shows the intensities of the electric field components and polarization distributions of the light fields formed directly after the depolarizer, the *q*-plate, and their combination. The further numerical analysis of the focusing of the formed vector light fields was performed using the well-known Richards–Wolf equations [17,40] (input field radius: 200 μm; numerical aperture of the focusing optical system: 0.15). In the case of the combination of elements (a depolarizer with a *q*-plate, *q* = ½), OV beams of the ±1 order are formed in the ±1 diffraction orders. The detailed analysis shows the formation of the ±1-order vortex phases in the *x*- and *y*-components of the field, and the ±2-order vortex phases in the *z*-component. The polarization states of the formed beams are orthogonal circular polarization, as in the case of Gaussian beams formed in the ±1 diffraction order using a single depolarizer.

Equation (14) shows that as the order (*q*) of the polarizing plate increases, the order of the formed vortex beams (*m* = 2*q*) also increases. However, high-order polarization plates are exotic, so various methods are used to create high-order CVBs [41]. It is also well known that higher-order CVBs can be formed using low-order *q*-plates and half-wave plates [42]. For example, the combination of a first-order *q*-plate, half-wave plate, and second-order *q*-plate converts the original *x*-linearly polarized light field into a third-order CVB, as follows:

(15) $$\begin{array}{l} E_{out}=J_{q=1}HJ_{q=1/2}E_{in}=\\ =\left[\begin{array}{cc} \cos\left(2\varphi \right) & \sin\left(2\varphi \right)\\ \sin\left(2\varphi \right) & -\cos\left(2\varphi \right) \end{array}\right]\left[\begin{array}{cc} 1 & 0\\ 0 & -1 \end{array}\right]\left[\begin{array}{cc} \cos\varphi & \sin\varphi \\ \sin\varphi & -\cos\varphi \end{array}\right]\left(\begin{array}{c} 1\\ 0 \end{array}\right)=\left(\begin{array}{c} \cos\left(3\varphi \right)\\ \sin\left(3\varphi \right) \end{array}\right), \end{array}$$

where *H* is the Jones matrix of a half-wave plate with a fast axis in the horizontal direction. These calculations can be applied for various numbers of such combinations. A CVB with a polarization order equal to the sum of the polarization orders of the CVBs formed by these *q*-plates individually is formed. This means that we can generate higher-order FPGs using lower-order *q*-plates.

**Figure 4:**
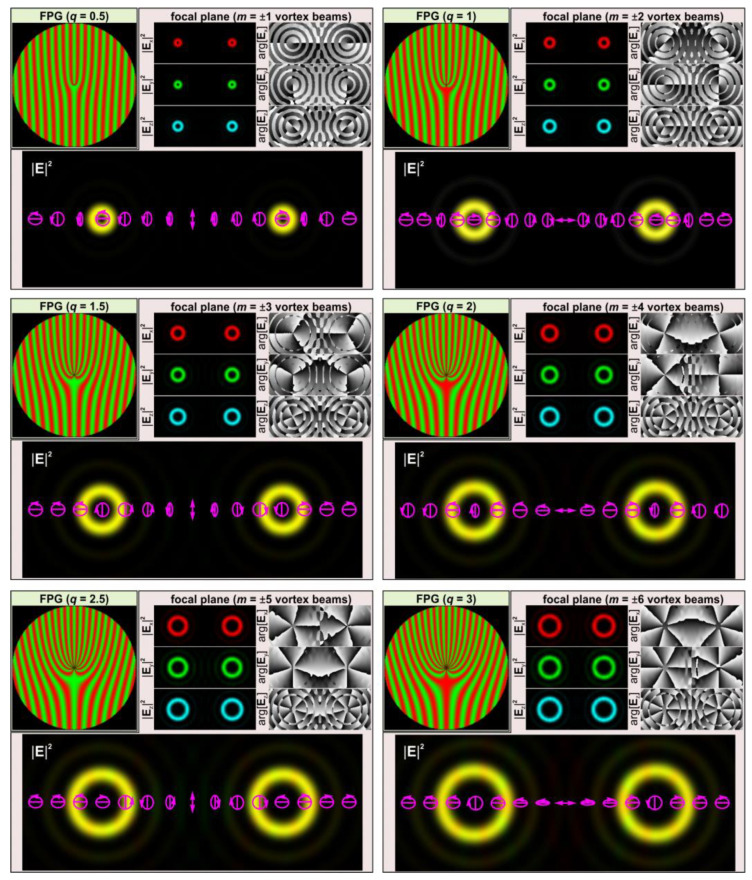
Focusing of light fields generated by FPGs of various orders. Three components of the electric field (red—*x*-component; green—*y*-component; and blue—*z*-component) and the total intensity/polarization distribution formed in the Fourier plane are shown. The arrows indicate local polarization directions. The ellipses with arrows are polarization ellipses. The size of the input field distributions is 200 × 200 μm.

We adapted these two approaches to create FPGs with orders from 2 to 12 simply using commercially available first- and second-order *q*-plates from Thorlabs Inc. [43] and half-wave plates. Figure 4 shows the amplitude and polarization profiles of a linearly polarized light field transformed by such combinations of elements. As with conventional amplitude or phase fork-shaped gratings, the focusing of the FPGs leads to the generation of optical vortex beams with opposite signs of topological charges in conjugate diffraction orders.

In our experiments, the light fields defined by Equation (13) were used for the laser patterning of thin films of NMLSs based on CGs. Previously, the holographic writing of fork-shaped gratings in CGs was studied based on the interference of Gaussian and first-order vortex beams with different polarizations [44]. In this case, fork-shaped gratings of the first order were created, allowing the formation of two vortex beams of the ±1st order outside the optical axis. In this work, we used light fields with the desired polarization distribution on the film with photosensitive material. The intensities of the *x*- and *y*-components of the fields defined by Equation (13) are defined as follows:

(16) $$\begin{array}{l} I_{x}(x,y)=\cos^{2}\left(\alpha x-2q\varphi \right)=\frac{1}{2}\left[1+\cos\left(2\alpha x-4q\varphi \right)\right],\\ I_{y}(x,y)=\sin^{2}\left(\alpha x-2q\varphi \right)=\frac{1}{2}\left[1-\cos\left(2\alpha x-4q\varphi \right)\right]\;. \end{array}$$

At the same time, it is well known that the intensity of the superposition of an inclined plane wave and an *m*th-order OV beam (exp(*im*ϕ)) is defined as follows:

(17) $$\begin{array}{l} I(x,y)={\left|\exp\left(i\alpha x\right)+\exp\left(im\varphi \right)\right|}^{2}=\\ =\left[\exp\left(i\alpha x\right)+\exp\left(im\varphi \right)\right]\left[\exp\left(-i\alpha x\right)+\exp\left(-im\varphi \right)\right]=\\ =2\left[1+\cos\left(\alpha x-m\varphi \right)\right]. \end{array}$$

From a comparison of Equations (16) and (17), it is clearly seen that the order of the formed fork-shaped grating and the frequency of the lines are doubled when the *q*-plate and the depolarizer are combined. This happens because, instead of a phase distribution, an amplitude distribution is actually formed (i.e., field components are expressed through cosine and sine functions but not through exponential functions).

Moreover, using the results of the works [45,46], it is possible to qualitatively predict the shape of the relief that will be formed in photosensitive materials. Taking into account the dependence of the light field defined by Equation (13) only on the transverse components, the following expression can be used to calculate the shape of the relief:

(18) $$\begin{array}{l} h(x,y)\propto \frac{\partial^{2}}{\partial x^{2}}{\left|E_{x}(x,y)\right|}^{2}+\frac{\partial^{2}}{\partial y^{2}}{\left|E_{y}(x,y)\right|}^{2}+\\ +\frac{\partial^{2}}{\partial xy}\left(E_{x}^{*}(x,y)E_{y}(x,y)+E_{y}^{*}(x,y)E_{x}(x,y)\right). \end{array}$$

**Figure 5:**
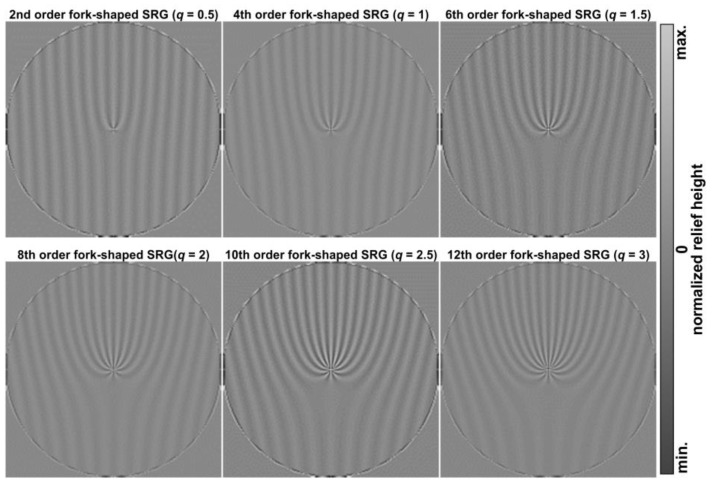
Predicted profiles of fork-shaped SRGs formed because of laser processing of photosensitive materials using FPGs with different orders.

Figure 5 shows the relief shape distributions calculated using Equation (18) for the light field defined by Equation (13). As can be seen, the relief corresponds to the fork-shaped SRG with the 4*q* order. Thus, during the diffraction of the incident beam on the relief formed in the CG (Figure 5), a pair of vortex beams of the *m* = ±4*q* order will be formed, which corresponds to doubling the vortex orders compared to the field focusing (13) shown in Figure 4. The major result of this analysis is the formation of fork-shaped gratings with a doubled order in the transverse field components. Namely, these distributions can be used for projection lithography techniques and the fabrication of fork-shaped SRGs in CGs.

## 3. Results

**Figure 6:**
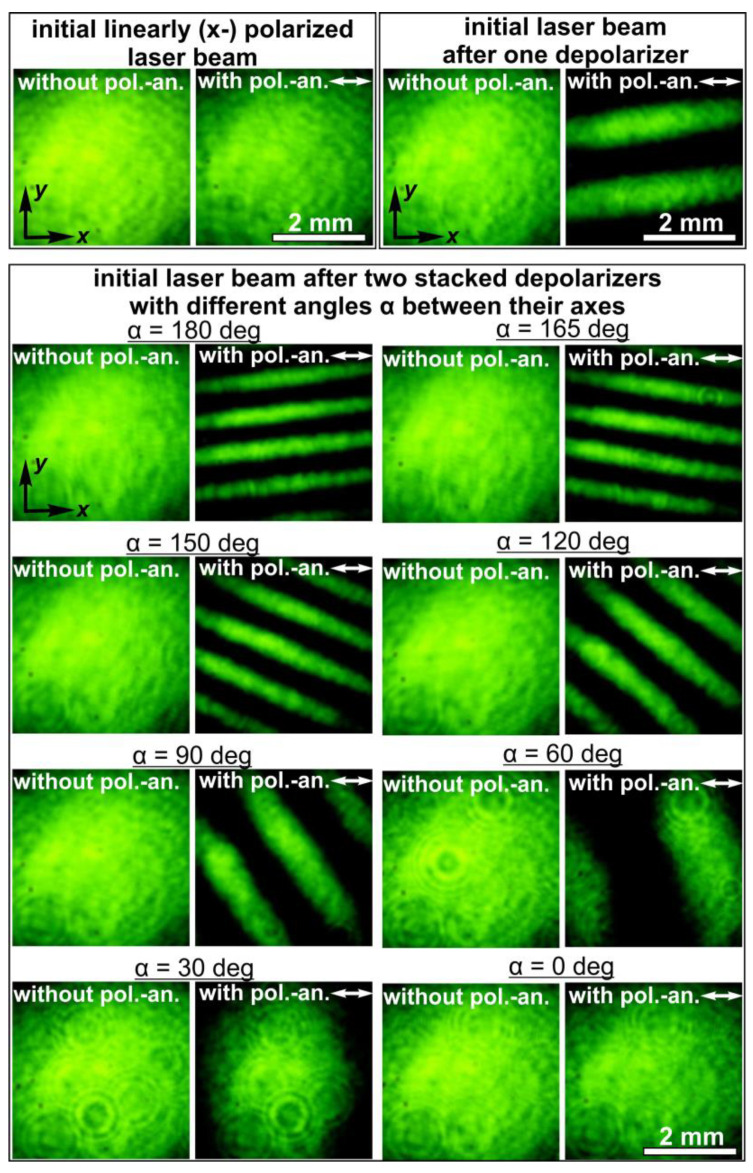
Intensity distributions of laser beams obtained with and without a polarizer–analyzer for the initial laser beam, the laser beam transformed by one depolarizer, and the laser beams transformed by two stacked depolarizers with different angles (α) between their axes.

To experimentally study the proposed approaches, two LCP depolarizers from Thorlabs Inc. (Newton, NJ, USA) [21] with the distribution of the fast-axis orientation shown in Figure 1b were used. In both depolarizers, the angle of the fast axis increases by 2 degrees across each consecutive 25 µm strip. In this case, *T* = 25 µm, θ_0_ = 2 degrees, and *d_dpe_* = π*T*/θ_0_ = 90*T* = 2250 µm directly after the element. The intensity profile of the laser beam transformed by these elements does not change and remains the same as that of the incident laser beam. The intensity distributions experimentally generated using two stacked depolarizers for various orientations and obtained with/without a polarizer–analyzer for various angles between the axes of the depolarizers are shown in Figure 6. These results are in good agreement with the performed theoretical analysis: the polarization is linear when the orientations of the stacked depolarizers coincide. The rotation of one of the elements leads to a change in the polarization modulation period. The minimal period of the shaped polarization modulation is obtained in the case of α = 180 degrees, and its value is half the modulation period obtained for a single depolarizer. In addition, there is no polarization modulation when α = 0 degrees; the transformed laser beam has a linear polarization in accordance with Equation (5).

**Figure 7:**
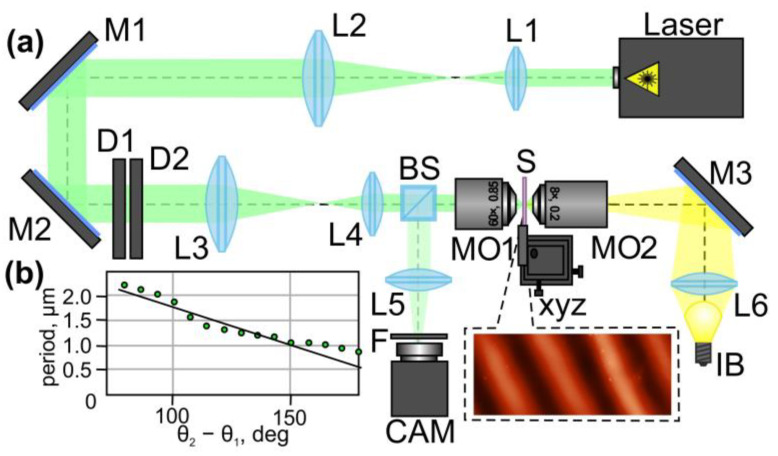
Laser printing of SRGs using two stacked depolarizers. (**a**) The optical setup used in the experiments. Laser is a solid-state laser (λ = 532 nm); L1, L2, L3, L4, L5, and L6 are spherical lenses (*f*_1_ = 25 mm; *f*_2_ = 250 mm; *f*_3_ = 150 mm; *f*_4_ = 25 mm; *f*_5_ = 150 mm; and *f*_6_ = 50 mm); M1, M2, and M3 are mirrors; D1 and D2 are two depolarizers; BS is a beam splitter; MO1 and MO2 are micro-objectives (NA = 0.85 and 0.2); S is a glass substrate with a thin film of NMLSs based on CGs; xyz is a three-axis (XYZ) translation stage; IB is a light bulb; F is a neutral-density filter; CAM is a ToupCam UCMOS08000KPB video camera. The inset shows an AFM image of a fabricated SRG. (**b**) Dependence of the period of the formed SRGs on the angle θ_2_ − θ_1_ between two depolarizers. The green circles indicate the experimental data. The black solid line is a linear approximation of the experimental data.

The optical setup used for the laser processing of NMLSs based on CGs is shown in Figure 7. In the experiments, we used a continuous-wave solid-state laser (wavelength λ: 532 nm; maximum output laser power (Pout): 90 mW) generating a linearly polarized Gaussian beam. The laser beam was first extended and collimated with two lenses (L1 and L2 (focal lengths of 25 and 250 mm)). Mirrors (M1 and M2) were used to direct the collimated beam onto a combination of depolarizers (D1 and D2). Lenses (L3 and L4 (focal lengths of 150 and 25 mm)) that make up a 4-f optical system were used to reduce the diameter of the formed laser beam. This was necessary to match the beam diameter and the entrance pupil diameter of the MO1 micro-objective (60×, NA = 0.85). The use of this 4-f optics system made it possible to form the same light field at the entrance pupil of the micro-objective directly behind the combination of depolarizers. The glass substrate (S) with a thin film of NMLSs based on CGs was mounted on the three-axis XYZ translation stage and located in the focal plane of the micro-objective MO1. For the illumination of the surface of the used glass substrate with CGs, we used a light bulb (IB), spherical lens (L6) (focal length of 50 mm), mirror (M6), and micro-objective (MO2) (8×, NA = 0.2). For the observation of the surface of the sample during laser processing, we used a beam splitter (BS), lens (L5) (focal length of 150 mm), and video camera (CAM) (TOUP-CAM UHCCD00800KPA; 1600 × 1200 pixels, with a pixel size of 3.34 μm). In order to simultaneously observe the surface of the sample and the formed light field, we used a neutral-density filter (F) to reduce the intensity of the light field.

In the experiments, the maximum output laser power was 80 mW. During the experiments, we rotated the second depolarizer (D2) relative to the first depolarizer (D1) to change the period of the polarization modulation of the converted laser beam. As mentioned above, a change in the angle between the axes of the depolarizers leads to a change in the modulation period of the generated periodically changing polarization distribution. The exposure time of the used sample for each of the studied angles of the rotation of the depolarizers relative to each other was 1 s. The inset in Figure 7a shows an atomic force microscopy (AFM) image of a section of one of the fabricated SRGs. The rotation of the second depolarizer led to an almost linear change in the period of the written SRGs (see Figure 7b). Thus, the experimentally obtained results are in good agreement with the theoretical predictions. The minimum period of the fabricated SRGs was submicron (approx. 0.95 µm) and was obtained for θ_2_ − θ_1_ = 180 degrees. The relief height of the fabricated SRGs was in the range from 50 to 500 nm, depending on the output laser power.

**Figure 8:**
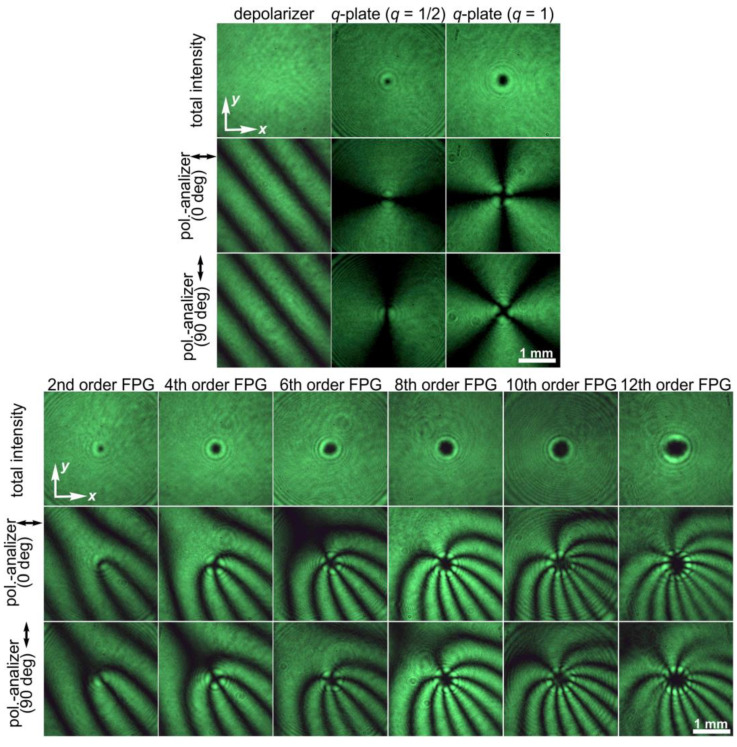
Intensity distributions of light fields obtained without and with a polarizer–analyzer for a linearly polarized Gaussian beam transformed by a depolarizer, a *q*-plate (*q* = 1/2), a *q*-plate (*q* = 1), and their combinations with half-wave plates, which allow the generation of FPGs of orders from 2 to 12. Two different orientations of the polarizer–analyzer were used.

**Figure 9:**
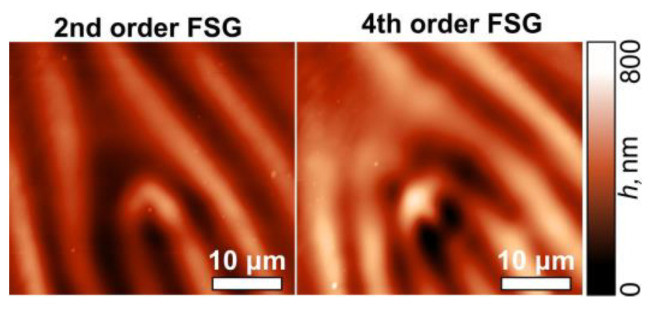
AFM images of the second- and fourth-order fork-shaped SRGs fabricated using a combination of a *q*-plate (*q* = ½ (**left**) and *q* = 1 (**right**)) and a depolarizer.

Figure 8 shows the experimentally obtained intensity distributions for two different orientations of the polarizer–analyzer, demonstrating the intensity distributions of the *x*- and *y*-components in accordance with Equation (16). Some of these combinations were used for the processing of the sample with a thin film of CGs and the fabrication of second- and fourth-order fork-shaped SRGs (see Figure 9). In this case, the optical setup used for the laser processing using two stacked depolarizers was slightly modified: lens L4 was removed, lens L3 was replaced with another lens with a focal length of 400 mm, and micro-objective MO1 was replaced with another micro-objective (40×, NA = 0.65). In this case, lens L3 and micro-objective MO1 make up a 4-*f* optical system for implementing a projection lithography setup. The structure of the desired polarization distributions is transferred to the used thin film of CGs.

**Figure 10:**
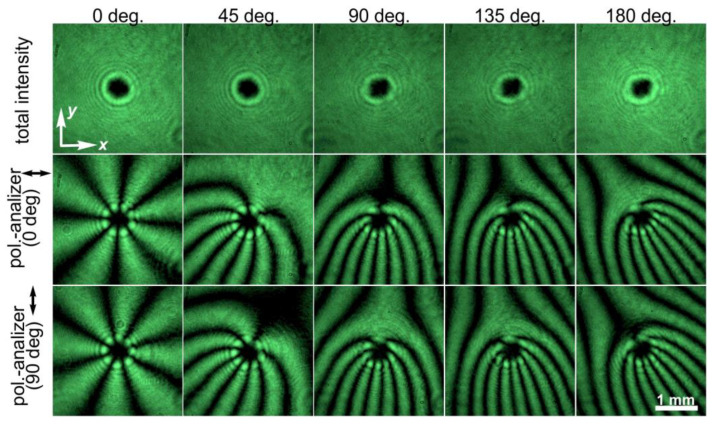
Intensity distributions of light fields obtained without and with a polarizer–analyzer for a linearly polarized Gaussian beam transformed by a combination of a *q*-plate (*q* = 1), a half-wave plate, a *q*-plate (*q* = 1), and two depolarizers. Various orientations of the depolarizer axes relative to each other are shown. Two different orientations of the polarizer–analyzer were used.

The next logical step is to study the possibility of controlling the period of FPGs formed using a depolarizer and *q*-plate. To achieve this, one can add a depolarizer, as in the case of the forming of linear gratings with different periods. In this case, the orientation of one of the depolarizers relative to the other elements in the combination allows one to change the period of the linear polarization modulation of the generated light field. The order of the formed FPGs can be controlled using combinations of *q*-plates and half-wave plates. The results obtained in the case of two *q*-plates of the order *q* = 1 and a half-wave plate located between them are shown in Figure 10. The rotation of one of the depolarizers leads to a change in the period of the polarization modulation: the minimum period is achieved for an angle of 180 degrees, as in the case of linear gratings formed using two stacked depolarizers. In the case of an angle of 0 degrees between the axes of the depolarizers, there is no polarization modulation of the original linearly polarized light field passing through these two stacked depolarizers. Because of this, in the output plane after all the elements, instead of forming an eighth-order HGT, only a fourth-order CVB order is formed.

## 4. Conclusions

In conclusion, we showed the possibility of using various combinations of polarizing optical elements, such as depolarizers, low-order *q*-plates, and half-wave plates, for the generation of linear and fork-shaped polarization gratings with modulated parameters. This approach made it possible to transform only the polarization distribution of the input linearly polarized laser beam, while the total amplitude distribution remained the same. We applied the formed polarization gratings for the direct laser patterning of thin films of NMLSs based on the CGs’ As_2_S_3_ and a-Se.

We showed the possibility of using stacked depolarizers for the implementation of the non-interferometric laser writing of linear SRGs. The control of the period of the SRGs was carried out by the simple rotation of one of the used depolarizers relative to another. The obtained results showed the possibility of the formation of SRGs with submicron periods (0.95 µm). The use of micro-objectives with larger magnification and numerical apertures should result in even smaller periods. The use of a larger number of stacked depolarizers could potentially also be used to further reduce the period of the fabricated gratings. In addition, the period can be reduced by using depolarizers with a smaller strip size or a larger increase in the angle of the fast axis across each consecutive 25 µm strip. In our opinion, the maximum angle in this case is 90 degrees. The proposed technique makes it possible to create more compact setups for the laser writing of SRGs and provides more convenient control of the period of the fabricated SRGs compared to interferometric approaches.

We also modified the proposed approach and used combinations of depolarizers and low-order *q*-plates (*q* = ½ and *q* = 1) to generate second- and fourth-order FPGs. The additional insertion of half-wave plates between the used *q*-plates made it possible to form FPGs with orders from 6 to 12. Control of both the frequency of the lines of the formed gratings and the number of their teeth was demonstrated. We applied the formed FPGs for the patterning of thin films of CGs with projection lithography. The manufacture of fork-shaped SRGs was successfully demonstrated. These results show the significant potential of using structured polarized light fields for the laser processing of such materials and can be adapted for the fabrication of other micro- and nano-optical elements.

## Data Availability

Data underlying the results presented in this paper are not publicly available at this time but may be obtained from the authors upon reasonable request.
